# The Mkx- TGF-β pathway induced the development of the myodural bridge complex in SD rats

**DOI:** 10.3389/fcell.2025.1639191

**Published:** 2025-08-14

**Authors:** Ji-Hang Li, Wen-Bin Jiang, Lu Zhang, Yun-Feng Liu, Yi-Tong Sun, Hua-Xun Lai, M. Adeel Alam Shah, Wei Ma, Chan Li, Campbell Gilmore, Jian-Fei Zhang, Hong-Jin Sui

**Affiliations:** ^1^Department of Anatomy, College of Basic Medicine, Dalian Medical University, Dalian, Liaoning, China; ^2^Department of Radiology, Dalian University Affiliated Xinhua Hospital, Dalian, Liaoning, China; ^3^Department of Anatomy, College of Basic Medicine, JiLin Medical University, Jilin, China; ^4^Medical School, St. George's University of London, London, United Kingdom

**Keywords:** CSF, Myodural Bridge Complex, MKX, TGF-β, developmental regulation

## Abstract

**Objective:**

The myodural bridge complex (MDBC) is a tendon-like structure highly conserved during vertebrate evolution, suggesting it plays an important physiological role. Substantial evidence indicates that the MDBC may contribute to cerebrospinal fluid (CSF) circulation by generating mechanical force. Studying its developmental process may offer new insights into CSF dynamics and lead to improved strategies for diagnosing and treating neurodegenerative diseases.

**Materials and Methods:**

This study utilized utilized lentiviral plasmids to either knockdown or overexpress the Mkx gene in newborn Sprague-Dawley rats (SD) rats, establishing three groups: control, overexpression group, and interference group. Suboccipital injections were performed at birth. Histological staining and qPCR were conducted at multiple time points to assess the morphological and genetic impacts of Mkx modulation on the development of the MDBC.

**Results:**

Transfection efficiency was confirmed by Green fluorescent protein (GFP) expression quantification, *in vivo* bioluminescent imaging, and Western blot validation in all experimental cohorts. Mkx knockdown exhibited diminished collagen fiber development accompanied by compensatory hyperplasia of occipital periosteum-derived fibrous tissues. Transcriptomic analysis revealed that Mkx overexpression upregulated tendon-related genes (Scx, Egr1) and downregulated myogenic regulators (Myod), with inverse expression patterns observed in knockdown models. Pathway gene analysis identified the TGF-β signaling cascade and associated mechanosensitive genes as central regulators of the MDBC.

**Conclusion:**

Mkx exerts bidirectional regulation on MDBC development by modulating the TGF-β signaling pathway. Overexpression of Mkx promotes collagen deposition and structural reinforcement in MDBC through coordinated molecular mechanisms: upregulating Scx/Egr1 expression, downregulating Myod, and inducing hyperplastic growth of deep fascial fibers in the rectus capitis dorsal minor muscle (RCDmi). Conversely, Mkx suppression maintains tissue integrity through three synergistic mechanisms: upregulating Myod expression, inducing MDBC fiber proliferation, and facilitating adaptive remodeling of the posterior atlanto-occipital membrane (PAOM). At the molecular level, Mkx coordinates differentiation processes through dynamic equilibrium of Scx/Egr1/Myod expression profiles while constructing regulatory networks that couple biomechanical-chemical signals via TGF-β pathway activation.

## 1 Introduction

Cerebrospinal fluid (CSF) circulation constitutes a fundamental mechanism for maintaining central nervous system homeostasis, facilitating metabolic waste clearance, nutrient transport, and neuroimmune modulation (Du et al., 2024). Classical perspectives attribute CSF hydrodynamic propulsion primarily to mechanical drivers including arterial pulsation (cardiac rhythm), respiratory dynamics, and postural variation (Jensen et al., 2025). Emerging research reveals the myodural bridge complex (MDBC), a unique anatomical structure at the craniocervical junction, formed by fibrous connections between suboccipital muscles and the spinal dura mater (SDM) (Zheng et al., 2017; Zheng et al., 2020), potentially contributes to CSF hydrodynamic regulation (Xu et al., 2021; Xu et al., 2016; Ma et al., 2021; Young et al., 2021; Li et al., 2022a; Yang et al., 2023). Notably, the MDBC is evolutionarily conserved across a wide range of vertebrate species, including reptiles (crocodiles, Brazilian tortoises, snakes), mammals, and certain fish (which exhibit MDB-like structures), suggesting fundamental physiological significance (Zheng et al., 2017; Young et al., 2020; Chen et al., 2025; Zhaohuangfu et al., 2019; Li et al., 2022b). Elucidating the molecular mechanisms governing MDBC development may provide critical insights into the structural and functional basis of CSF circulation.

**FIGURE 1:**
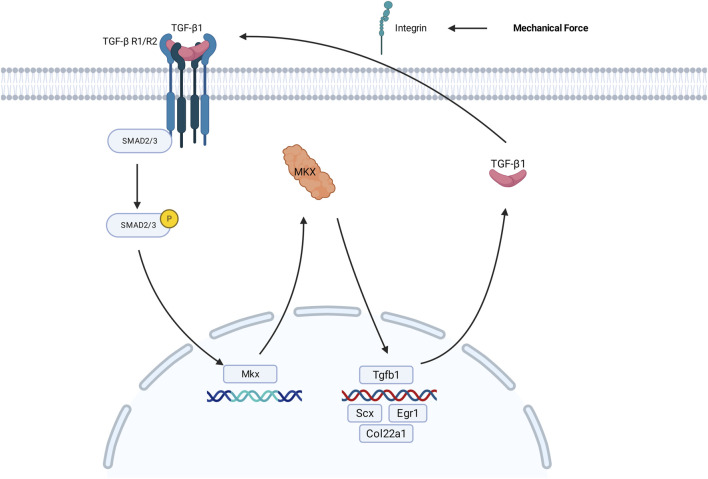
Schematic representation of Mkx-mediated regulatory mechanisms within the TGF-β signaling pathway during the development of tendon fibroblasts. Pathway activation initiates through ligand binding: TGF-β1 forms a complex with TβRI/II, subsequently inducing phosphorylation of SMAD2/3. Phosphorylated SMAD2/3 complex cooperatively activate transcriptional programs associated with ECM remodeling, including Mkx expression, through engagement with promoter-specific DNA motifs. The transcription factor MKX regulates transcriptional regulation of Scx, Egr1, Col22a1, and Tgfb1, thereby facilitating collagen biosynthesis and maintaining structural integrity in tendon tissues. Mechanical loading induces integrin-mediated liberation and activation of TGF-β1, which subsequently triggers activation of the TGF-β signaling pathway. TGF-β1, transforming growth factor-β1; TβRI/II,transforming growth factor-β Receptor 1/Receptor 2; SMAD2/3, essential signal transduction proteins in the TGF-β pathway; ECM, extracellular matrix; Mkx, Mohawk Homeobox gene; Tgfb1 gene encodes the TGF-β1 protein.

Studies have shown that MDBC is mainly composed of type I collagen fibers secreted by fibroblasts, and its anatomical location and structural features closely resemble those of tendinous connective tissues (Lai et al., 2021). These structural and developmental similarities suggest that key regulatory genes involved in tendon formation may also play critical roles in MDBC development. Figure 1 describes the central role of the TGF-β signaling pathway in regulating the morphogenesis of tendinous tissues (Havis et al., 2014; Liu et al., 2015; Dex et al., 2016; Kuo et al., 2008).

Activation of the pathway begins when extracellular bioactive TGF-β ligands, such as TGF-β1, bind to type I and type II surface receptors (RI/RII), leading to the formation of heteromeric receptor complexes. This interaction triggers the phosphorylation of SMAD2/3 (pivotal intracellular effectors of the TGF-β superfamily signaling pathway) and promotes their activation of downstream target genes within the cell (Goodier et al., 2016). The phosphorylated SMADs then form complexes that translocate to the nucleus, where they interact with other transcription factors to regulate the transcription of target genes, either promoting or repressing their expression (Tie et al., 2022). Notably, this pathway exhibits tissue-specific regulatory networks in its downstream effects.

The tendon-specific gene Mohawk Homeobox (Mkx) promotes ECM-related gene expression (Col22a1) through SMAD-dependent signaling, facilitating collagen cross linking and maturation to regulate tendon development and homeostasis (Havis et al., 2014; Liu et al., 2015; Dex et al., 2016). As a homeobox transcription factor, MKX protein binds specific promoter regions via its DNA-binding domain to direct mesenchymal stem cell tenogenic differentiation, upregulating target genes (Scx, Egr1) that enhance collagen biosynthesis and maintain tendon structural integrity (Lin et al., 2022; Takada et al., 2022; Lee et al., 2020; Havis and Duprez, 2020). As a target of Mkx, Scx activates Col1a1/Col1a2 expression to drive type I collagen secretion, establishing the biomechanical foundation for tendon function (Shukunami et al., 2006; Schweitzer et al., 2001). Simultaneously, the mechanical microenvironment plays an essential regulatory role in tendon development. EEgr1 rapidly responds to mechanical stress or injury stimuli by modulating tenocyte proliferation and ECM remodeling, dynamically adapting tissue biomechanical demands (Lejard et al., 2011). Studies demonstrate that integrins facilitate tendon development through mechanotransduction (Chatterjee et al., 2022; Mousavizadeh et al., 2020; Kronenberg et al., 2020), where Gtf2ird1 functions as a mechanosensor gene detecting tensile forces to promote cytoskeletal reorganization and cellular mechanoadaptation, synergistically activating TGF-β signaling with integrin-mediated mechanical transduction (Kayama et al., 2016). TGF-β ligands (TGF-β1) exist as latent precursors within the extracellular matrix (Xu et al., 2018). Research reveals that integrins transmit mechanical forces to the cytoskeleton via latent TGF binding proteins (LTBP) binding; applied tension stretches LTBP to release and activate latent TGF-β, thereby initiating TGF-β signaling cascades that drive tendon development (Shi et al., 2011).

In cellular differentiation regulation, certain genes exhibit remarkable specificity and cooperativity in their functions. For instance, Myod, a master regulator of myogenesis, shows downregulated expression during tendon differentiation while directing mesenchymal stem cells toward myogenic lineages (Wang et al., 2022), creating functional antagonism with Mkx-mediated tendogenesis to maintain dynamic equilibrium in cell fate determination. These gene-specific regulatory mechanisms provide critical insights for deciphering MDBC morphogenesis and functional homeostasis.

Recent studies have demonstrated that MDBC development is positively regulated by the TGF-β signaling pathway, with morphogenesis showing a strong correlation with TGF-β1 expression levels, suggesting that the pathway contributes to MDBC formation through ECM remodeling (Qin et al., 2024). A concurrent study identified integrin ITGA7 as a critical regulator of MDBC development, with suboccipital muscle-derived mechanical stress potentially promoting MDBC fiber differentiation/maturation through ITGA7-mediated mechanisms (Liu et al., 2024a). Developmental anatomical analyses in Sprague-Dawley (SD) rats further revealed that MDBC maturation begins around postnatal day 7 (Lai et al., 2021; LAI et al., 2022). Recent studies have demonstrated primitive reflexes mature by P5, signifying stabilization of spinal neural circuitry; and the P9 stage represents a preparatory phase for cervical proprioception and anti-gravity extension (Dupuis et al., 2024). Research identified postnatal days 7–14 as critical transitional phase for murine tendon maturation (FAN et al., 2022). The current study systematically investigated Mkx-TGF-β interactions in MDBC development through lentiviral-mediated Mkx modulation at critical stages (P5/P7/P9), analyzing pathway gene dynamics and MDBC morphogenetic mechanisms to advance understanding of CSF circulation physiology.

## 2 Materials and Methods

All animals used in research were obtained with permission from the Chinese Authorities for Animal Protection, and the study was approved by the Ethics Committee of Dalian Medical University. All experiments were performed in accordance with the guidelines and regulations of Dalian Medical University.

### 2.1 Experimental animals

This study was unable to calculate the sample size because there is no literature, to our knowledge, on the pathway of regulating MDBC pathway in SD Rats, and no preliminary data. This was an exploratory experiment. A total of 116 newborn SD rats were initially allocated for this experiment. After excluding subjects due to pre-weaning mortality, 86 valid samples were retained for analysis. The distribution across developmental stages (P5/P7/P9) and detailed exclusion criteria are summarized in Table 1. The study was conducted at three time points: days 5, 7, and nine post-birth (P5, P7, P9) with newborn SD rats. There were five groups at P7: Mkx-1, Mkx-2, Mkx-3 (three different interference silencing targets), Mkx+ (overexpression group), and NC (blank plasmid control group). Following the identification of silencing targets, P5 and P9 were categorized into three groups: Mkx- (confirmed interference silencing group), Mkx+, and NC. The experiments were repeated independently.

**TABLE 1:** Actual sampling records with data exclusion notes.

	P5			P7					P9		
Section	NC	MKX+	MKX-	NC	MKX+	MKX-1	MKX-2	MKX-3	NC	MKX+	MKX-
Masson	4	5	5	4	4	1	1	3	4	4	4
qPCR	3	3	3	3	3	0	0	3	3	3	3
WB	0	0	0	3	3	3	3	3	0	0	0
Total	81										

Note: Exclude invalid data such as deaths and operational errors.

### 2.2 Lentivirus preparation experiments

Mkx overexpression plasmids, knockdown plasmids and NC plasmids (scrambled sequence) were purchased from Shanghai Jikai Gene Technology Co., Ltd., including three knockdown plasmids: Mkx-RNAi (121051-2), Mkx-RNAi (121049-1), and Mkx-RNAi (121050-1). At least one of these RNA interference lentiviral constructs achieved a knockdown efficiency of over 60% at the mRNA level in target cells. The plasmids were subsequently amplified in bacterial culture, followed by plasmid extraction. They were then transfected into pre-cultured HEK 293T cells. Following 24 h of culturing the cells, they were observed under a fluorescence inverted microscope. The success of lentiviral transfection into 293T cells is determined by detecting the fluorescence intensity of GFP. After viral production, the lentiviral particles were harvested, precipitated, and concentrated to obtain a lentiviral stock for subsequent injection.

### 2.3 Lentivirus injection

Newborn P0 SD rats were euthanised via intraperitoneal injection of tribromoethanol solution at a dosage of 0.005 mL/g body weight. The external occipital protuberance was identified. Using a surgical ruler (graduations: 1 mm), a point 1 mm caudal to this landmark along the midsagittal line was measured and designated as the main reference point. At this reference point, bilateral injection sites were symmetrically marked by skin pen at 1 mm lateral to the midsagittal line (left and right), with positioning verified using a surgical ruler (graduations: 1 mm). An appropriate volume of lentiviral solution was drawn into an insulin syringe, aligned with the first injection site, and the needle was inserted vertically to a depth of 2 mm in a slow and controlled manner. At each site, 10 µL of lentiviral solution was injected gradually. Following each injection, the needle was left in place for 1 min to prevent backflow from the injection site. The procedure was then repeated at the second marked site. The success of the injections was assessed by checking for any spillage of the solution post-procedure. Post-injection, Newborn rats were labeled and returned to their dam for continued rearing. On the day of tissue harvesting, GFP expression was examined using a small animal imaging system to determine successful transduction.

### 2.4 Histological slices and staining

After lentivirus injection during the P0 period,the newborn SD rats at stages P5, P7, and P9 were euthanised with a 0.005 mL/g tribromoethanol solution. Transcardial perfusion was performed with warm saline (37), followed by 4% paraformaldehyde (PFA) fixation and stored in 4% PFA for 48 h. The rat specimens underwent decalcification in 10% EDTA solution for 4 weeks. Afterward, the samples were rinsed with water, dehydrated in a graded alcohol series, processed with xylene, and embedded in paraffin wax. The embedded specimens were sagittally sectioned (8 μm thickness) using a Leica Micro HM450 rotary microtome (Leica Micro HM450; Leica Microsystems GmbH, Wetzlar, Germany). Sections were then observed under an optical microscope, placed on glass slides, rehydrated, and stained with Masson’s trichrome (Shanghai yuanye Bio-Technology Co., Ltd.), using the same method for all sections. All the stained sections were analyzed using a Nikon research optical microscope, and the staining of sections was observed with a polarizing microscope (Olympus BH-2; Olympus Corp., Tokyo, Japan).

### 2.5 Calculation and statistical analysis of collagen volume fraction

Tissue regions were consistently selected from the posterior atlanto-occipital interspace (PAOiS), encompassing the spinal dura mater (SDM), the posterior atlanto-occipital membrane (PAOM), and portions of the rectus capitis dorsalis minor (RCDmi) muscle. ImageJ software was used to quantify the percentage of blue-stained collagen fibers relative to the total image area. Experimental data were statistically analyzed and graphically presented using GraphPad Prism 8.0. Results were expressed as Mean ± Standard Deviation (SD). A p-value of less than 0.05 was considered indicative of a statistically significant difference.

### 2.6 Western blot (WB) analysis

According to the experimental grouping requirements, after the newborn rats from different time periods in the lentivirus overexpression group, interference silence group and blank control group were euthanized, the suboccipital muscles were immediately isolated and sampled. Muscle bundles from RCDmi were isolated, excised *en bloc*, and snap-frozen in liquid nitrogen within 30 s post-excision. All procedures were completed within 5 min per rat to minimize RNA degradation. Protein concentrations were determined using the BCA protein assay method, with a uniform loading amount of 60 µg for each sample. Electrophoresis was started at 60 V, and the voltage was adjusted to 100 V once the samples entered the separating gel. Transfer was performed onto NC membranes at a constant current of 200 mA for 100 min. Blocking was done with a 5% milk solution on a room-temperature shaker for 1 h. The primary antibody incubation used was Mkx monoclonal antibody and the internal reference was Tubulinα polyclonal antibody, both purchased from Biogot Company. Signal detection was carried out using ECL chemiluminescence with band signals captured by Image Lab software. Data analysis was performed using GraphPad Prism 8.0 and ImageJ for statistical analysis and graphing, using ANOVA and Student's t-tests, with results presented as Mean ± SD.

### 2.7 Quantitative real-time PCR

The same tissue samples used for the Western blot experiments were employed for quantitative real-time PCR (qPCR). A portion of 10–20 mg from each sample was collected, and 500 µL of Buffer RL along with two small steel beads were added. Samples were homogenized using a cryogenic magnetic homogenizer until no visible tissue fragments remained. cDNA synthesis was performed using a reverse transcription kit (Biocompete Inc.), and qPCR was conducted using a commercial kit (Vazyme Biotech Co., Ltd.). Data analysis was performed using GraphPad Prism 8.0. Results were presented as mean ± standard deviation (Mean ± SD). Statistical significance between two groups was assessed using t-tests, with * indicating p < 0.05, ** indicating p < 0.01, and *** indicating p < 0.001.

## 3 Results

### 3.1 Lentivirus model validation

#### 3.1.1 Results of cell transfection

**FIGURE 2:**
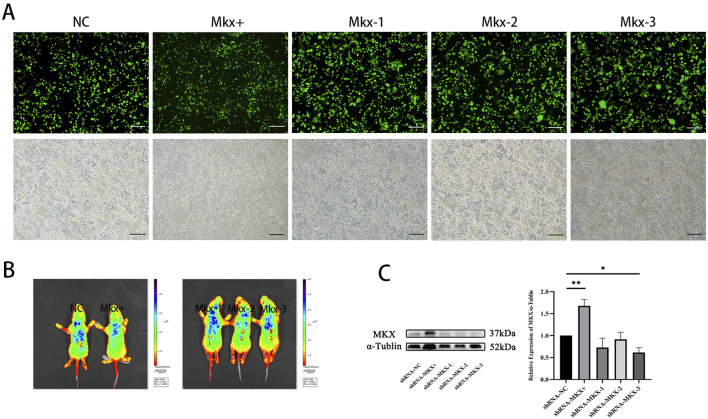
Fluorescence image of GFP expression in 293T cells 24 h post-lentiviral transfection. **(A)** Fluorescence images respectively represent the results of the NC, Mkx+, Mkx-1, Mkx-2, and Mkx-3 groups. Below, figures match the bright-field outcomes of the above images. All images share the same scale, set at 50 μm. **(B)** *In vivo* imaging results of GFP 7 days post localized lentiviral injection. From left to right, the NC Mkx+, Mkx-1, Mkx-2, and Mkx-3 groups. The blue areas denote the dispersion range of the lentivirus inside the body. **(C)** Western blotting analysis of MKX protein expression levels 7 days post-lentiviral injection. **(C)** shows the band pattern of MKX protein expression post-lentiviral injection,and illustrates the statistical analysis of MKX protein expression levels following lentiviral injection. *: P < 0.05, **: P < 0.01.

During lentivirus preparation, GFP fluorescence intensity was observed at 24 h post-transfection of 293T cells. In the NC group, Mkx+, and three Mkx-groups, abundant GFP green fluorescence expression was observed (Figure 2), suggesting successful gene transfection at the cellular level in each group.

#### 3.1.2 Results of In vivo imaging

At the P0 stage, lentivirus was injected into the suboccipital muscles of neonatal SD rats to induce Mkx expression. The distribution and expression levels of GFP fluorescence protein contained in the lentivirus were observed and analyzed using a small animal imaging system. The experimental results (Figure 2) indicated significant GFP fluorescence expression in all groups, including the NC, Mkx+ group, and the three Mkx-groups, suggesting successful gene transfection across all groups at the *in vivo* level.

#### 3.1.3 Western blot results

After 7 days of lentivirus injection, the results of each experimental group were compared with those of the control group with blank plasmid. The findings indicated a notable increase in Mkx expression in the suboccipital muscles of SD rats in the Mkx+ group (Figure 2), with this difference being statistically significant (p < 0.01).

Within the Mkx-groups, the Mkx-3 group showed a significant decrease in Mkx expression in the suboccipital muscles of SD rats, statistically significant (p < 0.05), demonstrating the highest gene knockdown efficiency in the Mkx-3 group. Compared to others, the Mkx-1 and Mkx-2 groups did not achieve statistically significant results, potentially indicating unstable knockdown efficiency at the target sites, which did not effectively reduce Mkx gene expression. Thus, these observations justify selecting the Mkx-3 group as the experimental group for Mkx-, confirming the efficacy and reliability of the experimental model. Meanwhile, the increased Mkx expression in the suboccipital muscles of the Mkx+ group further validated that the establishment of Mkx- and Mkx+ models was successful at the molecular level, through the selection and optimization of different targets.

### 3.2 Morphological development of the posterior atlanto-occipital interspace MDBC across different stages

Since the P7 stage is crucial for the development of the MDBC, SD rats at postnatal stages P5, P7, and P9 were selected to evaluate the impact of Mkx on this developmental process.

#### 3.2.1 Morphological observation of the posterior atlanto-occipital interspace MDBC at the P5 stage

**FIGURE 3:**
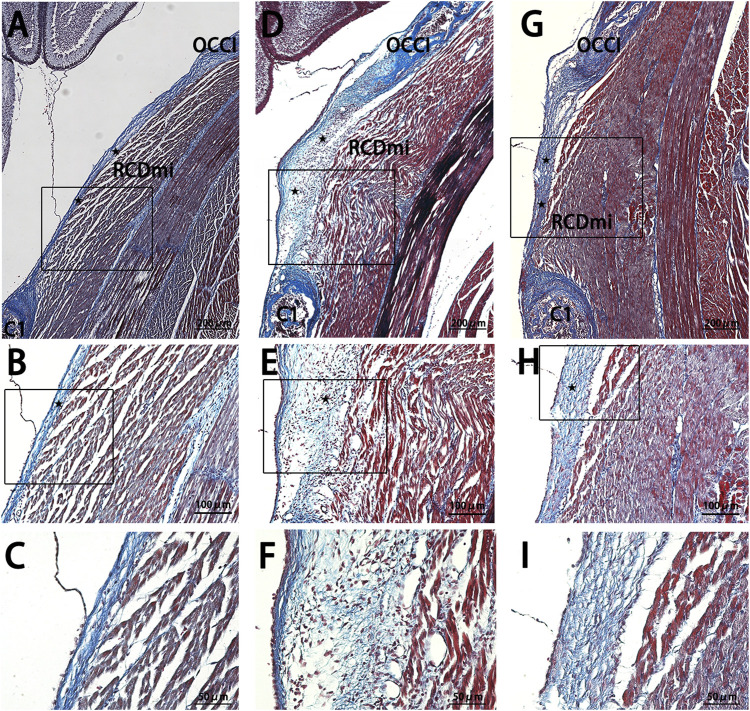
Masson stain results of the occipito-cervical sagittal sections during the P5 stage. OCCI: Occipital bone; C1: Posterior arch of atlas; RCDmi: Rectus capitis dorsal minor muscle; SC: Spinal cord; 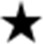:Atlanto-occipital posterior membrane; 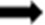:Ventral fibers; 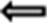:Dorsal fibers. **(A,B)**, and **(C)** represent the NC group Masson stain outcomes. **(D,E)**, and **(F)** belong to the Mkx+ group. **(G,H)**, and **(I)** are from the Mkx-group. Masson stain indicates a significant thickening of the atlanto-occipital posterior membrane in both Mkx+ and Mkx-groups, where the Mkx+ group displayed widespread collagen fibers in the RCDmi muscle, with a high presence of fibroblasts; For the Mkx-group, the dorsal fibers of the atlanto-occipital posterior membrane were denser, with irregular muscle patterns, and a reduced gap between the occipital bone and the atlas.

The MDBC in the PAOiS of P5-stage rats was examined using Masson’s trichrome staining (Figure 3). In the NC group, the PAOM displayed thin, densely packed, blue-stained collagen fibers. The RCDmi muscle adhered closely to the PAOM, with muscle fibers arranged in a compact and orderly fashion.

In the Mkx+ group, the PAOM was thicker, and the blue-stained collagen fibers spread extensively within the PAOiS, displacing the RCDmi muscle, with a looser muscle arrangement compared to the NC group. In the Mkx-group, the PAOM was markedly thicker with a higher density of collagen fibers, the fibers of the RCDmi muscle were irregularly arranged. In the Mkx+ and Mkx-groups, the fibers of the PAOM clearly split into two segments: one segment extended from the occipital bone to the atlas on the ventral side and integrated into the SDM (Figures 3D,E), while another segment extended from the atlas to the occipital bone on the dorsal side and merged with the PAOM (Figures 3G,H). In the Mkx-group, the fibers on the dorsal side of the PAOM were denser than those in the Mkx+ group (Figures 3F,I), suggesting a more mature development of the dorsal fibers.

#### 3.2.2 Morphological observations of the posterior atlanto-occipital MDBC at P7

**FIGURE 4:**
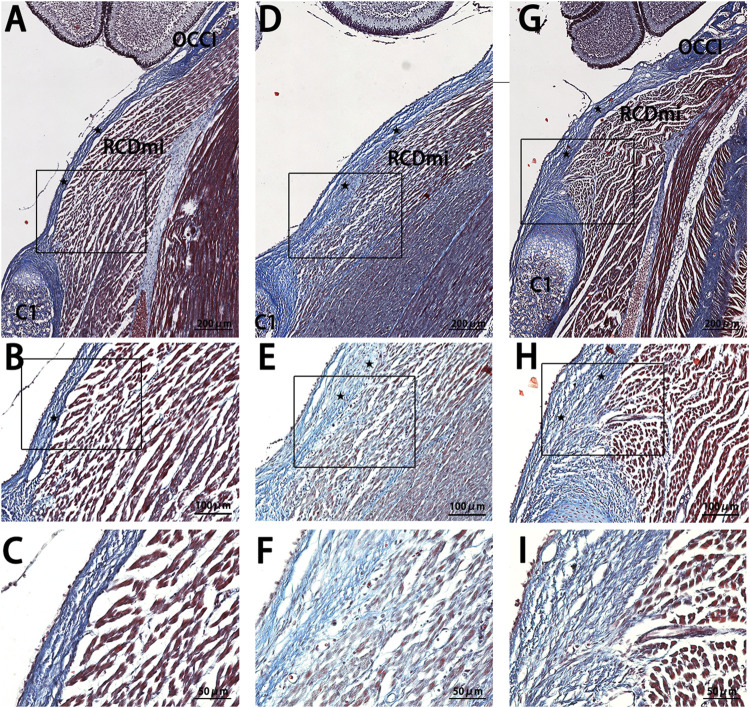
Masson stain results of the occipito-cervical sagittal sections at P7. OCCI: Occipital bone; C1: Posterior arch of the atlas; RCDmi: Rectus capitis dorsal minor muscle; 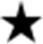 Atlanto-occipital posterior membrane. **(A,B)**, and **(C)** represent the NC group Masson trichrome staining outcomes. **(D,E)**, and **(F)** belong to the Mkx + group. **(G,H)**, and **(I)** are from the Mkx-group. Referring to the posterior atlanto-occipital membrane, Masson stain results indicate noticeable thickening in both Mkx+ and Mkx-groups. In the Mkx + group, the RCDmi muscle contains widespread collagen fibers, whereas in the Mkx-group, the posterior atlanto-occipital membrane’s dorsal fibers are denser, and the muscle arrangement is irregular.

In the P7-stage sagittal sections of the occipito-cervical area in SD rats (Figure 4), the NC group showed a thin posterior PAOM, with fibers from the RCDmi muscle seamlessly merging into it, presenting a loosely arranged yet regularly patterned muscle. The Mkx+ group continued to display thickening of the PAOM, with RCDmi muscle loosely arranged and regularly directed, containing numerous blue-stained collagen fibers. The Mkx-group showed even more pronounced thickening of the PAOM, with tightly arranged dorsal fibers and disordered arrangement of the RCDmi muscle, leading to a wavy course, and the gap between the occipital bone and atlas remained reduced.

#### 3.2.3 Morphological observations of the posterior atlanto-occipital MDBC at P9

**FIGURE 5:**
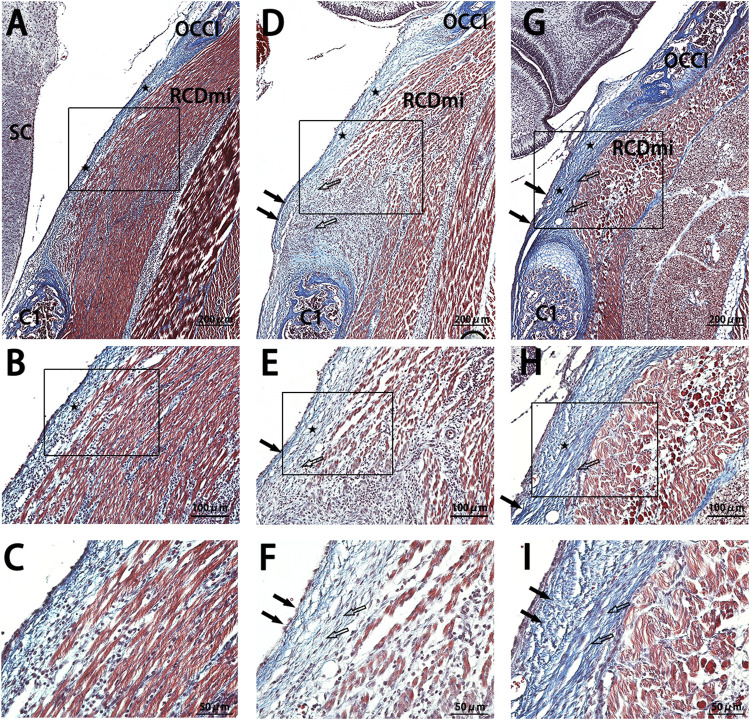
Masson stain results of the occipito-cervical sagittal sections at P 9. OCCI: Occipital bone; C1: Posterior arch of the atlas; RCDmi: Rectus capitis dorsal minor muscle; 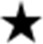
Atlanto-occipital posterior membrane. **(A,B)**, and **(C)** represent the NC group Masson’s trichrome staining outcomes. **(D,E)**, and **(F)** belong to the Mkx+ group. **(G,H)**, and **(I)** are from the Mkx-group. Masson stain shows significant thickening of the posterior atlanto-occipital membrane in both Mkx+ and Mkx-groups, with the Mkx + group RCDmi containing abundant collagen fibers and the membrane itself rich in fibroblasts; the Mkx-group also shows a significant thickening of the membrane.

In the sagittal sections of the occipito-cervical area at P9 (Figure 5), findings for the NC group resembled those from P7 (Figure 4), where the PAOM remained thin, and the RCDmi muscle had a regular course with loosely arranged muscles. For the Mkx+ group, the fibers extending from the atlas within the PAOM were loosely organized and disordered, with the nearby the RCDmi muscle on the ventral side of the membrane also being loosely arranged. In the Mkx-group, the continuous thickening of the PAOM and the loose arrangement of the RCDmi muscle were even more pronounced.

#### 3.2.4 Measurement of collagen volume fraction in the posterior atlanto-occipital Space

**FIGURE 6:**
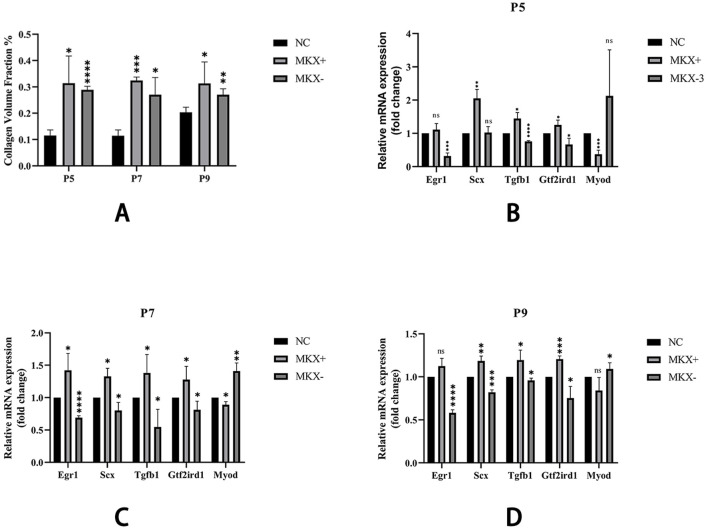
Collagen Volume Fraction results and qPCR results of gene expression across different groups. **(A)** Shown that within the same period, there are significant statistical differences between both Mkx+ and Mkx-compared to the NC group in the collagen fiber content. *: P < 0.05, **: P < 0.01, ****: P < 0.0001. **(B)** Levels of gene expression at P5. **(C)** Levels of gene expression at P7. **(D)** Levels of gene expression at P9. Tendon development-related genes: Egr1, Scx; Tendon development pathway gene: Tgfb1; Mechanosensory gene: Gtf2ird1; Muscle development gene: Myod. *: P < 0.05, **: P < 0.01, ***: P < 0.001, ****: P < 0.0001.

Masson stain was used to stain sections, turning the collagen fibers within the tissue blue. ImageJ was used to measure the collagen volume fraction of the blue-stained collagen fibers on Masson-stained slides, and this data was used for semi-quantitative analysis of collagen fiber expression. The semi-quantitative analysis results (Figure 6) show similar trends in collagen fiber content across the three sample groups at P5, P7, and P9 stages. Specifically, collagen fiber content was lowest in the PAOM of the NC group and highest in the Mkx+ group. The Mkx-group collagen fiber content, while lower than the Mkx+ group, was significantly higher than the NC group, showing significant statistical differences.

### 3.3 Variations in mkx expression result in transcriptional changes in related genes

Using qPCR analysis, the expression of various genes was compared in Mkx+ and Mkx-samples across the P5, P7, and P9 stages (Figure 6). Specifically, in the Mkx+ group, there was a decrease in the expression level of the muscle development gene Myod, and an increase in genes related to tendon development (such as Scx, Egr1), the tendon-related pathway gene Tgfb1, and the mechanosensory gene Gtf2ird1, indicating that Mkx favors tendon over muscle differentiation and development. Conversely, in the Mkx-group, while Myod expression increased, there was a decrease in the expression levels of the other mentioned genes. Moreover, the outcomes at P7 and P9 followed the same trends observed at P5.

## 4 Discussion

The MDBC, an evolutionarily conserved fibroconnective structure in vertebrates, potentially modulates cerebrospinal fluid dynamics through developmental mechanisms analogous to tendon differentiation (Zheng et al., 2017; Zheng et al., 2020; Xu et al., 2021; Xu et al., 2016; Ma et al., 2021; Young et al., 2021; Lai et al., 2021). Current evidence has established the TGF-β pathway as central to MDBC development, with integrins potentiating pathway activation through TGF-β1 upregulation (Qin et al., 2024; Liu et al., 2024a). The lack of identified key regulatory factors within the TGF-β pathway that govern MDBC development has hindered both morphogenetic investigations and functional studies on MDBC-CSF interactions.

### 4.1 Critical regulatory role of mkx in MDBC development

As an upstream regulatory gene, Mkx plays a critical role in tendon development (Nakamichi et al., 2016; Liu et al., 2024b). In the current study, Mkx-overexpressing groups demonstrated significant elevation of collagen volume fraction in the PAOiS, indicating enhanced fibrous content within the MDBC (Figure 6A). These findings align with previous experimental evidence (Tang et al., 2023). Mkx-suppression cohorts showed parallel but less pronounced fibrogenesis relative to overexpression groups, with control groups maintaining baseline fibrous architecture.

Studies have demonstrated three primary fiber sources in the PAOiS: the SDM, the periosteum located at the brim of the foramen magnum, and MDB contribute to the formation of the SDM (Zhuang et al., 2022). Mkx-overexpressing groups exhibited increased deep fascial fiber deposition from RCDmi muscle, indicating a key role of Mkx in promoting fibrogenesis and extracellular matrix accumulation (Figures 3F, 4F, 5F). Fibrous thickening in PAOiS has been shown to reduce dural compliance, impair CSF dynamics, and modulate craniocervical biomechanics (Yang et al., 2023; Song et al., 2023). In contrast, Mkx suppression cohorts displayed compensatory amplification of periosteal-derived fibrous components despite reduced collagen synthesis (Figures 3I, 4I, 5I). Transcriptomic analyses of Mkx tendons identified upregulated genes associated with muscular contraction, angiogenesis, and osteogenesis (Lin et al., 2022), automatically consistent with observed periosteal fibroproliferation under Mkx deficiency. This phenomenon likely represents a compensatory mechanism where alternative pathways are activated to preserve structural-functional integrity of the MDBC during Mkx inhibition. This inference is supported by relevant research findings, such as the discovery that functional loss of the TGF-β signaling pathway significantly leads to hyperplasia of the outer perichondrium (Sedes et al., 2022). In this experiment, under MKX knockdown conditions, significant fibrous proliferation originating from the occipital periosteum was observed, which is highly consistent with the aforementioned research discovery. We propose that this fibrous proliferation may constitute a direct compensatory response triggered by the suppression of TGF-β pathway activity regulated by MKX following its inhibition. These findings further indicate that MDBC development and structural maintenance involve sophisticated regulatory networks.

### 4.2 Mkx-mediated modulation of gene expression patterns in MDBC formation

Molecular analysis through qPCR revealed the regulatory effects of Mkx on MDBC-associated gene expression. Mkx-overexpressing specimens exhibited downregulation of the myogenic gene Myod (Figures 6B–D). This suggests preferential promotion of tendinous rather than muscular differentiation through Mkx overexpression. Upregulated expression of tendogenic markers Scx and Egr1 was observed (Figures 6B–D), aligning with established mechanisms (Liu et al., 2015; Havis and Duprez, 2020; Lejard et al., 2011). These results demonstrate functional conservation of Mkx regulation between MDBC and tendinous tissues. Conversely, Mkx suppression induced reciprocal transcriptional patterns: Myod upregulation accompanied by diminished tendogenic marker expression (Figures 6B–D). Reduced Scx/Egr1 levels under Mkx interference suggest impaired tendon differentiation and matrix development. These differential expression profiles underscore the essential role of Mkx in maintaining transcriptional equilibrium during MDBC development.

Furthermore, developmental stage-specific analyses (Figures 6B–D) demonstrated sustained Mkx-mediated gene regulation throughout the observation period, indicating its functional engagement from early MDBC morphogenesis stages with persistent effects until P9. These findings establish Mkx as a critical regulatory determinant during MDBC fiber development. Previous investigations have documented the essential role of Mkx in directing tenocyte lineage commitment and fibroblast differentiation (Liu et al., 2015; Takada et al., 2022; Chen et al., 2021). The experimental outcomes align with established molecular mechanisms, further supporting methodological validity.

### 4.3 Coordinated regulation of MDBC morphogenesis through TGF-β signaling and biomechanical forces

Figures 6 B–D demonstrate that Mkx overexpression significantly upregulated the TGF-β pathway core ligand gene Tgfb1 and mechanosensor gene Gtf2ird1, suggesting dual regulatory mechanisms of Mkx in MDBC development via TGF-β signaling: (1) Biochemical axis: Mkx overexpression elevates TGF-β1 expression to activate Smad2/3 phosphorylation cascades, driving transcriptional programs of MDBC developmental genes and enhancing collagen crosslinking and maturation (Liu et al., 2015; Dex et al., 2016; Guerquin et al., 2013). (2) Mechanotransduction axis: Mkx may amplify cellular mechanosensitivity by coordinating integrin-LTBP interactions, translating mechanical tension into LTBP deformation to release and activate latent TGF-β precursors, thereby potentiating pathway activity. Experimental evidence confirms that mechanical stress facilitates latent TGF-β release via integrin-LTBP axis, a process reinforced through TGF-β autoregulatory loops (Shi et al., 2011; ZHEN et al., 2021). The coordinated upregulation of Tgfb1 and Gtf2ird1 implies that Mkx-TGF-β signaling establishes dynamic regulatory networks through integrated mechanical-chemical coupling in MDBC morphogenesis.

The central regulatory role of Mkx-TGF-β axis in MDBC development provides novel insights into anatomical-physiological mechanisms underlying CSF dynamics. Numerous studies indicate that dysregulated cerebrospinal fluid dynamics constitutes a significant pathological factor in neurodegenerative diseases (Chuang et al., 2025; Tanaka et al., 2025; Jin et al., 2025). As a mechanotransduction hub at the suboccipital muscle-spinal dura mater, MDBC generates driving forces for CSF flow dynamics (Xu et al., 2021; Xu et al., 2016; Ma et al., 2021; Young et al., 2021; Li et al., 2022a; Yang et al., 2023). Consequently, elucidating the regulatory mechanisms of the Mkx-TGF-β axis in neurodegenerative models may offer novel insights into disease progression. Subsequent investigations will prioritize identifying downstream effectors of the Mkx-TGF-β axis within MDBC models of neurodegeneration. This approach will refine the molecular circuitry of this signaling axis and deepen understanding of its association with neurodegenerative pathology.

Notably, this study encountered methodological constraints: unquantified lentiviral titers, excessive dependence on rodent models, and inadequate sampling across developmental stages. Future studies should employ spatial transcriptomic sequencing of human MDBC tissues, humanized *in vitro* biomechanical simulation systems, and multi-timepoint longitudinal analyses to validate the crosstalk mechanisms of these pathways and expand their clinical translational potential.

## 5 Conclusion

This study demonstrates that Mkx plays a central regulatory role in MDBC development through modulation of the TGF-β pathway. Mkx overexpression enhances collagen deposition and structural reinforcement in MDBC by upregulating tendon-related genes (Scx, Egr1), suppressing myogenic gene (Myod), and stimulating hyperplastic growth of deep fascial fibers in the RCDmi muscle. Under Mkx suppression, structural integrity is preserved via compensatory mechanisms including MDBC fiber expansion from occipital periosteum, Myod upregulation, and adaptive thickening of the PAOM, thereby disrupts the development of MDBC. Mechanistically, Mkx coordinates MDBC differentiation by maintaining transcriptional equilibrium among Myod, Scx, and Egr1, ensuring proper development. Furthermore, the Mkx-TGF-β axis establishes a dynamic regulatory network through mechanical-chemical coupling, collectively governing structural maturation and functional integrity of MDBC. These findings reveal that Mkx, as a core gene, dynamically regulates MDBC development through the TGF-β signaling pathway.

## Data Availability

The data presented in the study are deposited in the Figshare database with DOI: https://doi.org/10.6084/m9.figshare.29597261.v1.
